# Fatigue burden and patient attribution across remission states in inflammatory bowel disease: a longitudinal cohort study

**DOI:** 10.1093/crocol/otag109

**Published:** 2026-09-24

**Authors:** Arun Swaminath, Jakob Saidman, Jeremy Polman, Laura Durbin, Rachel Maggi, Troy Laurence, Keith Sultan

**Affiliations:** Division of Gastroenterology, Lenox Hill Hospital, New York, NY 10025, United States; Division of Gastroenterology, Lenox Hill Hospital, New York, NY 10025, United States; Division of Gastroenterology, Lenox Hill Hospital, New York, NY 10025, United States; Division of Gastroenterology, Lenox Hill Hospital, New York, NY 10025, United States; Division of Gastroenterology, Lenox Hill Hospital, New York, NY 10025, United States; Division of Gastroenterology, Lenox Hill Hospital, New York, NY 10025, United States; Division of Gastroenterology, Northshore University Hospital, Manhasset, NY 11030, United States

**Keywords:** fatigue, IBD, inflammatory bowel disease, ulcerative colitis, Crohn’s disease

## Abstract

**Background and aims:**

Fatigue is common in inflammatory bowel disease (IBD), but prior work has largely focused on symptomatic disease activity rather than progressively objective remission states. We evaluated the relationship between fatigue and hierarchical remission depth, repeated changes in remission status, and patients’ attribution of fatigue to IBD.

**Methods:**

Adults with confirmed IBD completed PROMIS Fatigue surveys at 2 tertiary-care centers. PROMIS Fatigue was analyzed continuously and as clinically significant fatigue (T-score ≥55). Remission was classified hierarchically as no remission, physician-global-assessment (PGA)-defined clinical remission, endoscopic remission, or histologic remission. Visit-level repeated measures were analyzed using a linear mixed-effects model. First-visit logistic regression evaluated clinically significant fatigue after adjustment for anxiety, depression, anemia, and IBD subtype; corticosteroid exposure was added in a sensitivity model. Attribution analyses included only explicit Yes or No responses.

**Results:**

We surveyed 481 patients, contributing 900 surveys. PGA-defined clinical, endoscopic, and histologic remission remained associated with lower mean PROMIS Fatigue T-scores than no remission (adjusted differences −3.68, −3.45, and −3.27 points; all *P* ≤ .001). No remission remained associated with clinically significant fatigue after adjustment for anxiety, depression, anemia, IBD subtype (OR 2.75, 95% CI, 1.83-4.14), and for corticosteroid exposure (OR 2.34, 95% CI, 1.52-3.58). Attribution of fatigue to IBD differed by remission status (*P* < .000000001). Findings remained significant when objective remission was restricted to colonoscopy within 180 days of PROMIS completion (adjusted OR 2.23, 95% CI, 1.42-3.49).

**Conclusion:**

Remission was associated with lower fatigue burden, and patients in objective remission were less likely to attribute fatigue to IBD. These observational findings support assessing inflammatory disease control early in the evaluation of fatigue while also evaluating sleep, mood, anemia, pain, medications, and other non-inflammatory contributors.

Key Messages

- Across repeated measurements, all remission categories were associated with lower PROMIS Fatigue scores than no remission.
- Among participants providing an explicit Yes or No attribution response, patients not in remission were substantially more likely to attribute fatigue to IBD.
- The association between no remission and clinically significant fatigue persisted after adjustment for anxiety, depression, anemia, IBD subtype, and corticosteroid exposure.

## Introduction

Patients with inflammatory bowel disease (IBD), including ulcerative colitis (UC) and Crohn’s disease (CD), experience relapsing-remitting gastrointestinal symptoms and extraintestinal manifestations. Fatigue is common but is not specific to active intestinal inflammation. It may reflect anemia, medication effects, malnutrition, sleep disturbance, chronic pain, mood disorders, psychosocial stressors, or combinations of these factors.^1–6^

Fatigue affects patients with both active and inactive IBD, supporting an association with inflammatory activity while also demonstrating that fatigue frequently persists despite clinical quiescence. Much of the existing literature is cross-sectional, uses a single fatigue measurement, or defines disease activity using symptoms alone. These approaches may not capture within-person variation or distinguish symptomatic remission from more objective endoscopic and histologic remission.^7^

Several studies have incorporated longitudinal data on fatigue in the IBD. Klusmann et al. followed a Dutch IBD cohort over 9 years and reported a correlation between disease activity scores and fatigue severity.^8^ A 2-year longitudinal study of Canadian patients using a patient reported symptom questionnaire similarly found higher fatigue among patients with higher symptom burden, though approximately 20% of patients without symptoms had persistently high fatigue.^9^ Among clinically inactive patients, fatigue was correlated to depression, low quality of life, and female sex.^10^^,^^11^

In the present study, we evaluated fatigue across multiple time points in a U.S.-based IBD cohort. The study extends prior work by examining mutually exclusive hierarchical remission states, incorporating repeated observations within patients, and evaluating whether patients’ attribution of fatigue to IBD differs across remission states.

## Methods

### Study population

We conducted a longitudinal survey study of adults (≥18 years) with confirmed IBD (CD or UC) receiving care at 2 tertiary referral centers (2019-2023). Participants provided written informed consent and completed questionnaires in English during outpatient clinic or procedural follow-up visits. The study was approved by the Northwell Health Institutional Review Board.

### Measures and clinical definitions

Fatigue was assessed using the PROMIS Fatigue Short Form 7a with a 7-day recall period. PROMIS Fatigue was analyzed as a continuous T-score and categorically. Fatigue severity was categorized as none (<55), mild (55-60), moderate (61-70), or severe (>70), and clinically significant fatigue was defined as T-score ≥55. Participants were additionally asked whether they attributed fatigue to their underlying IBD. Because this item was added after early participant feedback, attribution analyses included only participants providing an explicit Yes or No response; “N/A—not fatigued” and missing responses were excluded from attribution denominators.^12–14^

Remission depth at each survey was determined from the electronic medical record and assigned as a mutually exclusive hierarchical category. Physician-global-assessment (PGA)-defined clinical remission reflected the treating physician’s global assessment with patient agreement. Endoscopic remission was defined as absence of visible inflammation on the most recent endoscopy report available at the time of evaluation. Histologic remission was defined as absence of chronic inflammation on routine histopathology from the most recent colonoscopy. Histologic remission required clinical and endoscopic remission; endoscopic remission required clinical remission and endoscopic remission without histologic remission; PGA-defined clinical remission indicated clinical remission without endoscopic or histologic remission.

Demographics, IBD subtype, medication exposures, and comorbidities were abstracted from the medical record. Anxiety, depression, and anemia were identified using ICD-10 codes documented before or at the first survey. CRP, fecal calprotectin, sleep disturbance, chronic pain, and standardized gastrointestinal symptom measures were not systematically available in the analytic dataset.

### Statistical analysis

PROMIS Fatigue T-scores were linked to remission depth at each evaluation. Cross-sectional analyses summarized continuous T-0scores and categorical fatigue severity. A linear mixed-effects model with a patient-level random intercept evaluated visit-level PROMIS Fatigue T-score differences across remission categories while accounting for repeated observations; no remission was the reference category, and IBD subtype was included as a covariate. No imputation was performed, and analyses used complete cases for the variables required by each model. PROMIS Fatigue T-scores were available for all 900 surveys. The mixed-effects analysis included 896 surveys with a classifiable remission status and CD or UC subtype.

Among patients with more than one survey, we also examined clinically interpretable transitions from no remission to any remission and from any remission to no remission. Within-person PROMIS Fatigue T-scores before and after transition were compared using paired analyses, and mean differences were reported with bootstrap 95% confidence intervals. These transition analyses were considered complementary to the mixed-effects model.

The previously planned directional-alignment analysis was treated as exploratory because it did not account for the full repeated-measures structure. The mixed-effects model was used as the principal longitudinal analysis. All tests were 2-sided with statistical significance defined as *P* < .05.

First-visit logistic regression evaluated clinically significant fatigue (PROMIS Fatigue T-score ≥55). The adjusted model included no remission, anxiety, depression, anemia, and IBD subtype. Because corticosteroid exposure may represent a treatment effect, marker of disease severity, or intermediate factor associated with active disease, a sensitivity model additionally adjusted for corticosteroid use. Analyses involving subtype were restricted to participants classified as CD or UC. The first-visit multivariable analyses included 474 participants with complete covariate data and a CD or UC diagnosis. Fatigue-attribution analyses included 349 participants who provided an explicit Yes or No response; N/A and missing responses were excluded from attribution denominators. A categorical remission-status-by-subtype interaction was evaluated separately.

Because timing of objective assessments relative to PROMIS completion was not prespecified in the original dataset, we performed a focused chart review of the 105 patients initially classified as being in endoscopic or histologic remission at the first survey. We recorded the colonoscopy and biopsy dates closest in time to the first PROMIS survey, calculated the time separating the objective assessment from PROMIS completion, and reviewed intervening clinical encounters for evidence of a change in disease status. The objective findings and intervening clinical record were used to corroborate remission status at enrollment. Identified classification errors were corrected before repeating the analyses. A sensitivity analysis restricted objective remission to patients whose colonoscopy was within 180 days of PROMIS completion.

## Results

### Baseline characteristics

We longitudinally surveyed 481 patients, resulting in 900 surveys. Mean age was 43 years and 46% were female. The cohort included 271 patients with CD and 205 with UC; a small number had indeterminate or missing subtype classification. At the first survey, 157 patients had clinically significant fatigue and 324 did not (Table 1). Corticosteroid use was more common among fatigued participants (35.0% vs 18.8%; *P* = .00016), whereas biologic, small-molecule, and immunomodulator exposure did not differ significantly. Explicit Yes/No fatigue-attribution responses were available for 349 participants; N/A and missing responses were excluded from attribution percentages. Comorbidity comparisons are shown in Figure S1.

**Table 1:** Baseline demographic and clinical characteristics of the first-visit cohort, stratified by clinically significant fatigue.

*N*	157	324
**Male, *n* (%)**	72 (45.9%)	184 (56.8%)
**Female, *n* (%)**	84 (53.5%)	139 (42.9%)
**Age, mean ± SD**	42.4 ± 15.3	42.0 ± 16.3
**Age, median (IQR)**	40.0 (29.5-51.5)	38.0 (29.0-55.0)
**CD, *n* (%)**	82 (52.2%)	189 (58.3%)
**UC, *n* (%)**	73 (46.5%)	132 (40.7%)
**IND, *n* (%)**	0 (0.0%)	2 (0.6%)
**BMI, median (IQR)**	24.8 (22.3-28.2)	25.5 (22.2-28.8)
Fatigue T-score, mean ± SD*	62.2 ± 4.9	45.0 ± 6.8
**Fatigue attributed to IBD: Yes, *n/N* (%)**	96/127 (75.6%)	75/222 (33.8%)
**Fatigue attributed to IBD: No, *n/N* (%)**	31/127 (24.4%)	147/222 (66.2%)
**Biologic, Yes**	86 (54.8%)	190 (58.6%)
**IMM, Yes**	12 (7.6%)	38 (11.7%)
**Small Molecule, Yes**	8 (5.1%)	7 (2.2%)
Corticosteroids, Yes*	55 (35.0%)	61 (18.8%)
Anxiety*	21 (13%)	20 (6.2%)
Depression*	24 (15%)	20 (6.2%)
Anemia*	30 (19%)	34 (10.6%)

*p<0.05

Fatigue severity differed by remission category (Figure 1A; *P* = 8.05 × 10^−5^), with severe fatigue concentrated among patients without remission or with PGA-defined clinical remission. Severe fatigue was not observed in endoscopic or histologic remission. In the mixed-effects model, PGA-defined clinical remission, endoscopic remission, and histologic remission were each associated with lower mean PROMIS Fatigue T-scores than no remission (adjusted differences −3.68 [95% CI, −4.89 to −2.47], −3.45 [−5.39 to −1.50], and −3.27 [−5.16 to −1.37], respectively).

**Figure 1:**
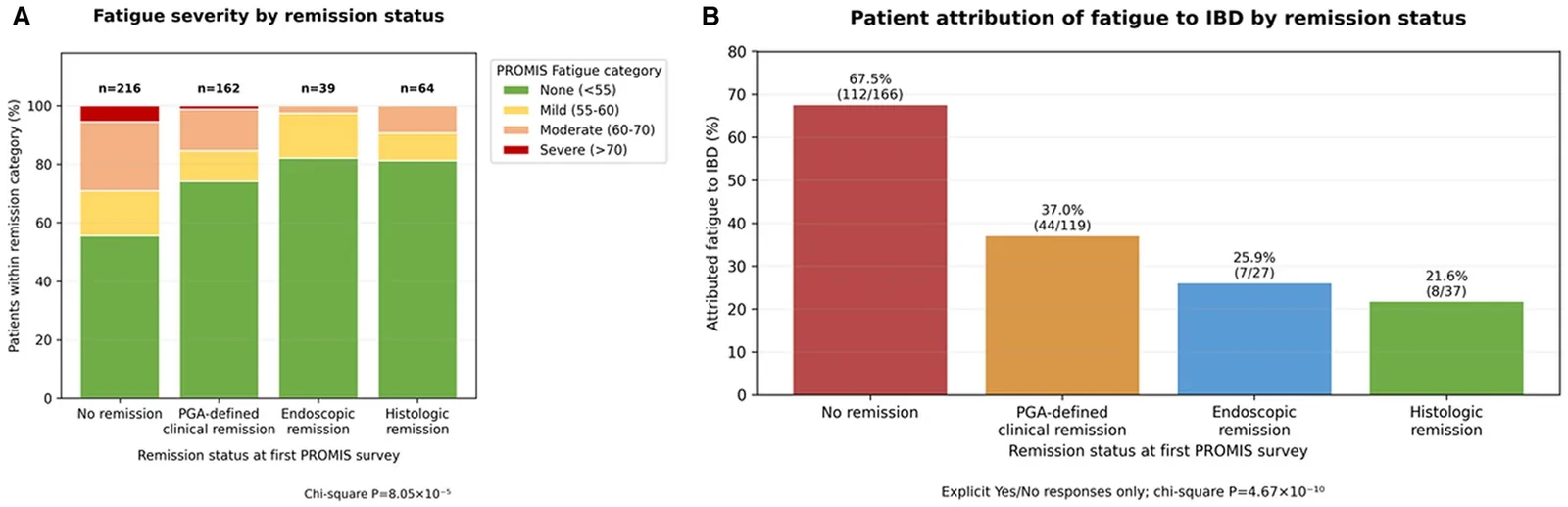
Fatigue severity and patient attribution by remission status. (A) PROMIS Fatigue severity categories were none (<55), mild (55-60), moderate (60-70), and severe (>70). (B) Percentage of participants explicitly attributing fatigue to IBD; N/A and missing responses were excluded. PGA, physician global assessment.

**Figure 2:**
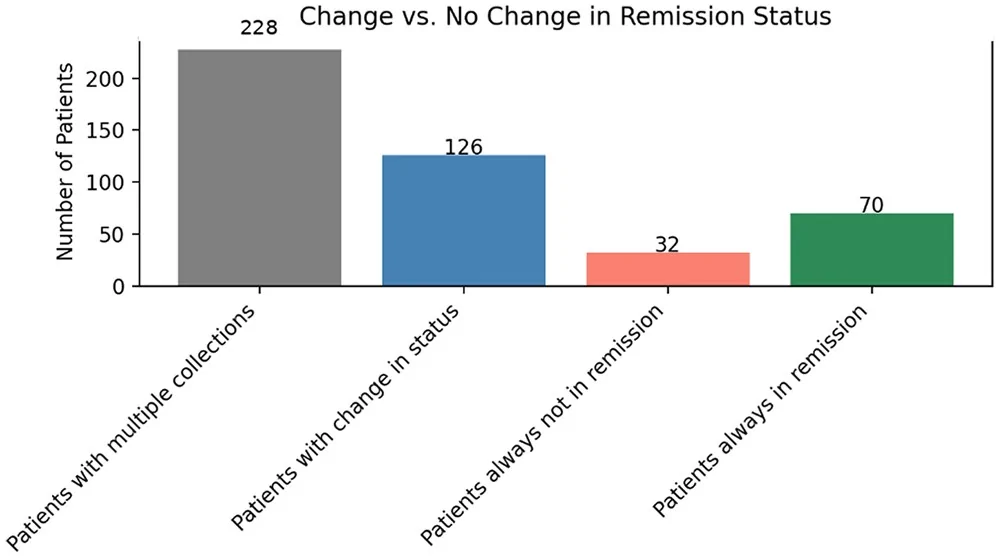
Within-person PROMIS Fatigue T-score changes among patients transitioning from no remission to any remission and from remission to no remission. Error estimates and confidence intervals are reported in the results.

Anxiety, depression, and anemia were more prevalent among fatigued participants. Not in remission remained associated with clinically significant fatigue after adjustment for anxiety, depression, anemia, and subtype (OR 2.75, 95% CI, 1.83-4.14). In a sensitivity model additionally adjusting for corticosteroid exposure, no remission remained significant (OR 2.34, 95% CI, 1.52-3.58), and corticosteroid use remained independently associated with fatigue. The categorical remission-status-by-subtype interaction was not statistically significant (*P* = .141). Principal mixed-effects and logistic-regression estimates are summarized in Table 2, with detailed results in Tables S1 and S2. Subtype analyses are provided in Table S4.

**Table 2:** Principal mixed-effects and multivariable logistic-regression results. Mixed-effects estimates represent adjusted mean differences in PROMIS Fatigue T-score relative to no remission and include a patient-level random intercept. The adjusted logistic model evaluated clinically significant fatigue, defined as PROMIS Fatigue T-score ≥55, and included anxiety, depression, anemia, and IBD subtype. The corticosteroid sensitivity model additionally included corticosteroid exposure. The 6-month timing sensitivity analysis restricted endoscopic and histologic remission to patients whose colonoscopy was within 180 days of PROMIS completion.

Analysis	Estimate	95% CI	*P*-value
**Mixed model: PGA-defined clinical remission vs no remission**	−3.68 PROMIS points	−4.89 to −2.47	<.000001
**Mixed model: Endoscopic remission vs no remission**	−3.45 PROMIS points	−5.39 to −1.50	.00051
**Mixed model: Histologic remission vs no remission**	−3.27 PROMIS points	−5.16 to −1.37	.00074
**Adjusted logistic model: No remission**	OR 2.75	1.83 to 4.14	.0000011
**Corticosteroid sensitivity model: No remission**	OR 2.34	1.52 to 3.58	.00010
**Six-month timing sensitivity: No remission**	OR 2.23	1.42 to 3.49	.00050

Abbreviations: CI, confidence interval; OR, odds ratio; PGA, physician global assessment.

Among participants providing an explicit Yes or No attribution response, attribution of fatigue to IBD differed by remission status (Figure 1B; *P* = 4.67 × 10^−10^). Fatigue was attributed to IBD by 112/166 (67.5%) participants without remission, 44/119 (37.0%) with PGA-defined clinical remission, 7/27 (25.9%) with endoscopic remission, and 8/37 (21.6%) with histologic remission.

In our cohort, 228 patients contributed repeated surveys (Figure 2). Among 78 patients transitioning from no remission to any remission state, mean PROMIS Fatigue T-score decreased from 54.3 to 51.0 (mean change −3.31 points; bootstrap 95% CI approximately −5.26 to −1.24). Among 48 patients transitioning from remission to no remission, mean PROMIS Fatigue T-score increased from 50.5 to 52.4 (mean change 1.61 points; 95% CI, −1.06 to 4.30).

Among the 103 patients in objective remission, the median time separating colonoscopy and PROMIS completion was 223 days (IQR 84-392); 46 had colonoscopy within 180 days. When objective remission was restricted to this 6-month interval, no remission remained associated with clinically significant fatigue (adjusted OR 2.23, 95% CI, 1.42-3.49). Additional chart-review and sensitivity results are provided in Table S3.

## Discussion

To our knowledge, this is among the first longitudinal studies to evaluate fatigue across mutually exclusive PGA-defined clinical, endoscopic, and histologic remission states and the first to combine this approach with patient attribution of fatigue to IBD. The mixed-effects analysis demonstrated lower fatigue in each remission category compared with no remission. The estimates did not establish a perfectly stepwise decline from clinical through histologic remission, and the observational design does not establish that achieving deeper remission will itself resolve fatigue.^15–18^

Attribution of fatigue to IBD also differed by remission status. After excluding N/A and missing responses, participants without remission were substantially more likely to attribute fatigue to IBD than those in clinical, endoscopic, or histologic remission. This single-item attribution measure cannot capture the extent to which inflammatory and non-inflammatory factors contribute simultaneously. Nevertheless, the finding supports a practical distinction: persistent fatigue in objective remission should prompt evaluation for sleep disturbance, mood disorders, anemia, chronic pain, medication effects, nutritional deficiencies, deconditioning, and psychosocial stressors.^19–26^

Advanced therapy exposure was not associated with fatigue. Corticosteroid use was independently associated with clinically significant fatigue and partially attenuated the no-remission estimate. This association should be interpreted cautiously because corticosteroids may contribute to sleep disruption or other symptoms, may identify patients with more severe inflammatory activity, or may lie partly on the pathway between active disease and fatigue. IBD subtype was not independently associated with fatigue, and adjustment for UC versus CD did not materially change the no-remission association.^27^

Several limitations warrant emphasis. PGA-defined clinical remission was not based on PRO2, PRO3, HBI, SCCAI, or another validated symptom index and may have misclassified disease activity. CRP and fecal calprotectin were not systematically available, limiting biomarker validation of the PGA-defined clinical-remission group. The timing between endoscopic assessment and PROMIS Fatigue completion limits causal and temporal interpretation. It would have been possible for disease activity to change between clinical assessment and observed association. However, focused chart review identified only 3 classification errors, review of intervening encounters identified one documented flare, and the findings remained significant when objective remission was restricted to colonoscopy within 6 months of PROMIS completion. Although our adjusted analyses accounted for anxiety, depression, anemia, IBD subtype, and corticosteroid exposure, other recognized contributors to fatigue—including sleep disturbance, chronic-pain, nutritional, or psychosocial measures—were not systematically measured. Residual confounding from these unmeasured factors is possible. Attribution was measured with a single item and was unavailable or not applicable for some participants. Repeated surveys occurred at variable intervals, and the observational design precludes causal inference.^28^^,^^29^

In conclusion, remission was associated with lower fatigue burden, and patients in objective remission were less likely to attribute fatigue to IBD. These findings support confirming inflammatory disease control early in the evaluation of fatigue, while recognizing that persistent fatigue is frequently multifactorial. Among patients in objective remission, evaluation and treatment should focus on non-inflammatory contributors rather than assuming that further escalation of IBD therapy will improve fatigue.

## Supplementary Material

otag109_Supplementary_Data

## Data Availability

Data available upon request to the corresponding author.

## References

[1] Rogler G , Singh A, Kavanaugh A, Rubin DT. Extraintestinal manifestations of inflammatory bowel disease: current concepts, treatment, and implications for disease management. Gastroenterology. 2021;161:1118-1132. 10.1053/j.gastro.2021.07.042 PMC8564770 34358489

[2] Canakis A , Qazi T. Sleep and fatigue in IBD: an unrecognized but important extra-intestinal manifestation. Curr Gastroenterol Rep. 2020;22:8. 10.1007/S11894-020-0746-X 32002666

[3] Christensen KR , Ainsworth MA, Steenholdt C, et al Fatigue is a systemic extraintestinal disease manifestation largely independent of disease activity, chronicity, and nutritional deficiencies in inflammatory bowel disease on biologics. Scand J Gastroenterol. 2022;57:1051-1057. 10.1080/00365521.2022.2060049 35412932

[4] Nocerino A , Nguyen A, Agrawal M, Mone A, Lakhani K, Swaminath A. Fatigue in inflammatory bowel diseases: etiologies and management. Adv Ther. 2020;37:97-112. 10.1007/S12325-019-01151-W PMC6979464 31760611

[5] Argollo M , Gilardi D, Peyrin-Biroulet C, Chabot JF, Peyrin-Biroulet L, Danese S. Comorbidities in inflammatory bowel disease: a call for action. Lancet Gastroenterol Hepatol. 2019;4:643-654. 10.1016/S2468-1253(19)30173-6 31171484

[6] Truyens M , Lernout H, De Vos M, Laukens D, Lobaton T. Unraveling the fatigue puzzle: insights into the pathogenesis and management of IBD-related fatigue including the role of the gut-brain axis. Front Med (Lausanne). 2024;11:1424926. 10.3389/FMED.2024.1424926 PMC11252009 39021817

[7] D’Silva A , Fox DE, Nasser Y, et al Prevalence and risk factors for fatigue in adults with inflammatory bowel disease: a systematic review with meta-analysis. Clin Gastroenterol Hepatol. 2022;20:995-1009.e7. 10.1016/j.cgh.2021.06.034 34216824

[8] Klusmann B , Fleer J, Tovote KA, et al Trajectories of fatigue in inflammatory bowel disease. Inflamm Bowel Dis. 2021;27:1919-1930. 10.1093/IBD/IZAB007 33769489

[9] Graff LA , Clara I, Walker JR, et al Changes in fatigue over 2 years are associated with activity of inflammatory bowel disease and psychological factors. Clin Gastroenterol Hepatol. 2013;11:1140-1146. 10.1016/j.cgh.2013.03.031 23602816

[10] Banovic I , Gilibert D, Cosnes J. Crohn’s disease and fatigue: constancy and co-variations of activity of the disease, depression, anxiety and subjective quality of life. Psychol Health Med. 2010;15:394-405. 10.1080/13548501003759155 20677078

[11] Villoria A , García V, Dosal A, et al Fatigue in out-patients with inflammatory bowel disease: prevalence and predictive factors. PLoS One. 2017;12:e0181435. 10.1371/JOURNAL.PONE.0181435 PMC5531543 28749985

[12] Feagan BG , Sandborn WJ, Sands BE, et al Qualitative and psychometric evaluation of the PROMIS®-fatigue SF-7a scale to assess fatigue in patients with moderately to severely active inflammatory bowel disease. J Patient Rep Outcomes. 2023;7:115. 10.1186/S41687-023-00645-0 PMC10645698 37962770

[13] Azevedo S , Oliveira MM, Nogueira P, Lopes AI. Clinical usefulness of patient-reported-outcome-measurement information system in pediatric Crohn’s disease: a cross-sectional study. Health Qual Life Outcomes. 2024;22:1-11. 10.1186/s12955-024-02330-2 PMC11686960 39736608

[14] Norton C , Czuber-Dochan W, Bassett P, et al Assessing fatigue in inflammatory bowel disease: comparison of three fatigue scales. Aliment Pharmacol Ther. 2015;42:203-211. 10.1111/APT.13255 25989464

[15] Holten KA , Bernklev T, Opheim R, et al Fatigue in patients with inflammatory bowel disease in remission one year after diagnosis (the IBSEN III study). J Crohns Colitis. 2025;19:1-9. 10.1093/ECCO-JCC/JJAE170 PMC12001332 39527064

[16] Loftus EV , Rubin DT, Louis E, et al Improvement in inflammatory bowel disease questionnaire items: fatigue, depression, anxiety, and bowel urgency in patients with Crohn’s disease treated with upadacitinib in phase 3 trials. Abstract Su1863. Presented at: DDW 2024; May 18-21, 2024; Washington, DC. PMC11345973 39193409

[17] Kamp KJ , Clark-Snustad K, Barahimi M, Lee S. Relationship between endoscopic and clinical disease activity with fatigue in inflammatory bowel disease. Gastroenterol Nurs. 2022;45:21-28. 10.1097/SGA.0000000000000600 PMC8820259 35020629

[18] Borren NZ , Long MD, Sandler RS, Ananthakrishnan AN. Longitudinal trajectory of fatigue in patients with inflammatory bowel disease: a prospective study. Inflamm Bowel Dis. 2021;27:1740-1746. 10.1093/IBD/IZAA338 PMC8528141 33367749

[19] Gong SS , Fan YH, Lv B, Zhang MQ, Xu Y, Zhao J. Fatigue in patients with inflammatory bowel disease in Eastern China. World J Gastroenterol. 2021;27:1076-1089. 10.3748/WJG.V27.I11.1076 PMC7985735 33776374

[20] Davis SP , Chen DG, Crane PB, Bolin LP, Johnson LA, Long MD. Influencing factors of inflammatory bowel disease-fatigue: a path analysis model. Nurs Res. 2021;70:256-265. 10.1097/NNR.0000000000000517 PMC8231668 33935213

[21] Czuber-Dochan W , Ream E, Norton C. Review article: description and management of fatigue in inflammatory bowel disease. Aliment Pharmacol Ther. 2013;37:505-516. 10.1111/APT.12205 23311461

[22] van Erp LW , Roosenboom B, Komdeur P, et al Improvement of fatigue and quality of life in patients with quiescent inflammatory bowel disease following a personalized exercise program. Dig Dis Sci. 2021;66:597-604. 10.1007/S10620-020-06222-5 32239380

[23] Rozich JJ , Holmer A, Singh S. Effect of lifestyle factors on outcomes in patients with inflammatory bowel diseases. Am J Gastroenterol. 2020;115:832-840. 10.14309/AJG.0000000000000608 PMC7274876 32224703

[24] Keightley P , Reay RE, Pavli P, Looi JCL. Inflammatory bowel disease-related fatigue is correlated with depression and gender. Australas Psychiatry. 2018;26:508-513. 10.1177/1039856218772245 29737197

[25] You L , Guo N, Wang T, et al Effects of aromatherapy on fatigue, quality of sleep and quality of life in patients with inflammatory bowel disease: a feasibility study. Complement Ther Clin Pract. 2022;49:101648. 10.1016/j.ctcp.2022.101648 35994795

[26] Lamers CR , De Roos NM, Heerink HH, Van De Worp-Kalter LA, Witteman BJM. Lower impact of disease on daily life and less fatigue in patients with inflammatory bowel disease following a lifestyle intervention. Inflamm Bowel Dis. 2022;28:1791-1799. 10.1093/IBD/IZAC027 PMC9713506 35212382

[27] Skjellerudsveen BM , Skoie IM, Dalen I, Grimstad T, Omdal R. The effect of biological treatment on fatigue in inflammatory bowel disease: a systematic review and meta-analysis. Drugs. 2023;83:909-921. 10.1007/S40265-023-01888-3 PMC10284989 37219801

[28] Kamp KJ , Hawes SE, Tse CS, et al IBD Qorus. Concordance and discordance between patient-reported remission, patient-reported outcomes, and physician global assessment. Inflamm Bowel Dis. 2023;29:1255-1262. 10.1093/IBD/IZAC206 PMC10393067 36179129

[29] Aass Holten KI , Bernklev T, Opheim R, et al Fatigue in patients with newly diagnosed inflammatory bowel disease: results from a prospective inception cohort, the IBSEN III study. J Crohns Colitis. 2023;17:1781-1790. 10.1093/ECCO-JCC/JJAD094 PMC10673818 37279652

