# Supplementary material for: Fatigue burden and patient attribution across remission states in inflammatory bowel disease: a longitudinal cohort study
Source: Crohns Colitis 360. 2026 Sep 24;8(4):otag109. doi: 10.1093/crocol/otag109 (PMC13649359; doi:10.1093/crocol/otag109)
Supplement: Supplementary file 1 [file otag109_supplementary_data.docx]

**Supplementary Material**

Fatigue Burden and Patient Attribution Across Remission States in Inflammatory Bowel Disease: A Longitudinal Cohort Study

**Supplementary Table S1. Linear mixed-effects model of PROMIS Fatigue T-score.**

| **Comparison** | **Adjusted mean difference in PROMIS Fatigue T-score** | **95% CI** | **P value** |
| --- | --- | --- | --- |
| PGA-defined clinical remission vs no remission | −3.68 | −4.89 to −2.47 | <0.001 |
| Endoscopic remission vs no remission | −3.45 | −5.39 to −1.50 | <0.001 |
| Histologic remission vs no remission | −3.27 | −5.16 to −1.37 | <0.001 |
| Ulcerative colitis vs Crohn disease | 0.93 | −0.71 to 2.58 | 0.266 |

The model included 896 usable surveys and a patient-level random intercept to account for repeated PROMIS surveys from the same participant. No remission was the reference category for remission status, and Crohn disease was the reference category for IBD subtype. Negative estimates indicate lower mean PROMIS Fatigue T-scores relative to the reference group. CI, confidence interval; PGA, physician global assessment.

**Supplementary Table S2. Fully adjusted logistic-regression sensitivity model for clinically significant fatigue.**

| **Variable** | **Adjusted odds ratio** | **95% CI** | **P value** |
| --- | --- | --- | --- |
| No remission | 2.34 | 1.52 to 3.58 | <0.001 |
| Anxiety | 1.87 | 0.84 to 4.16 | 0.122 |
| Depression | 2.21 | 1.03 to 4.73 | 0.042 |
| Anemia | 2.09 | 1.18 to 3.71 | 0.011 |
| Ulcerative colitis vs Crohn disease | 1.19 | 0.78 to 1.79 | 0.419 |
| Corticosteroid use | 1.88 | 1.17 to 3.03 | 0.009 |

The outcome was clinically significant fatigue, defined as PROMIS Fatigue T-score ≥55. All variables shown were included simultaneously in the model (N=474). Any remission, absence of anxiety, absence of depression, absence of anemia, Crohn disease, and no corticosteroid use were the reference categories. Odds ratios greater than 1 indicate higher odds of clinically significant fatigue. Corticosteroid exposure was included as a sensitivity covariate because it may reflect treatment effects, active or more severe disease, or an intermediate factor. CI, confidence interval.

**Supplementary Table S3. Focused chart review and six-month procedure-timing sensitivity analysis.**

| **Measure** | **Result** |
| --- | --- |
| Patients initially reviewed | 105 |
| Classification errors identified and corrected | 3 |
| Documented intervening clinical flare | 1 |
| Patients remaining in endoscopic or histologic remission | 103 |
| Median time separating colonoscopy and first PROMIS survey | 223 days |
| Interquartile range | 84 to 392 days |
| Full range | 8 to 1,568 days |
| Colonoscopy within 90 days of PROMIS | 29 patients |
| Colonoscopy within 180 days of PROMIS | 46 patients |
| Colonoscopy within 365 days of PROMIS | 73 patients |
| Total patients in six-month sensitivity cohort | 423 |
| Fatigue severity by remission status | P=0.00031 |
| Fatigue attribution by remission status | P=3.4×10⁻⁷ |
| Adjusted OR for clinically significant fatigue: no remission | 2.23 (95% CI 1.42 to 3.49) |
| Adjusted-model P value | 0.00050 |

Focused chart review included patients initially classified as being in endoscopic or histologic remission at the first PROMIS survey. The objective assessment closest in time to PROMIS completion and the intervening clinical record were used to corroborate remission status. The six-month sensitivity analysis retained endoscopic and histologic remission only when colonoscopy was within 180 days of PROMIS completion. The adjusted model included anxiety, depression, anemia, IBD subtype, and corticosteroid exposure. CI, confidence interval; OR, odds ratio.

**Supplementary Table S4. Clinically significant fatigue by remission status and IBD subtype.**

**Panel A. Subtype-stratified descriptive results**

| **IBD subtype** | **Remission status** | **N** | **Clinically significant fatigue, n (%)** |
| --- | --- | --- | --- |
| CD | No remission | 115 | 45 (39.1%) |
| CD | PGA-defined clinical remission | 98 | 23 (23.5%) |
| CD | Endoscopic remission | 26 | 6 (23.1%) |
| CD | Histologic remission | 31 | 8 (25.8%) |
| UC | No remission | 98 | 49 (50.0%) |
| UC | PGA-defined clinical remission | 63 | 19 (30.2%) |
| UC | Endoscopic remission | 11 | 1 (9.1%) |
| UC | Histologic remission | 33 | 4 (12.1%) |

**Panel B. Formal remission-status-by-subtype interaction test**

| **Test** | **Likelihood-ratio χ²** | **Degrees of freedom** | **P value** |
| --- | --- | --- | --- |
| Categorical remission status × IBD subtype | 5.45 | 3 | 0.141 |

Clinically significant fatigue was defined as PROMIS Fatigue T-score ≥55. The analysis included 475 patients with a CD or UC diagnosis and a classifiable first-visit remission status. The formal interaction treated remission status as a categorical variable rather than assuming a linear stepwise relationship across remission categories. The interaction was not statistically significant, indicating insufficient evidence that the relationship between remission status and fatigue differed between CD and UC. Small endoscopic-remission subgroup sizes limit precision. CD, Crohn disease; UC, ulcerative colitis.

**Supplementary Figure S1. Anxiety, depression, and anemia by fatigue status.**

Prevalence of anxiety, depression, and anemia among participants with clinically significant fatigue (PROMIS Fatigue T-score ≥55) versus those with T-score <55.
